# Supplementary material for: Unmet Needs of Parents of Children with Urea Cycle Disorders
Source: Children (Basel). 2022 May 12;9(5):712. doi: 10.3390/children9050712 (PMC9140128; doi:10.3390/children9050712)
Supplement: Supplementary file 1 [file children-09-00712-s001.zip › children-1671590-supplementary.pdf]

ABOUT YOU BEING A PARENT OF CHILD WITH A RARE DISEASE

[SOCIODEMOGRAPHIC CHARACTERISTICS]

Below are some of questions about you as a parent of a child with a rare disease. Please tick only one box per question. Tick the box that best describes you.

Are you a mother or father of a child with a rare disease?

☐1 Biological/adoptive mother or stepmother

☐2 Biological/putative/adoptive father or stepfather

☐8 I do not know/I am not sure.

☐9 I would rather not like to say.

What is your age?

________ years

☐8 I do not know/I am not sure.

☐9 I would rather not like to say.

In what country do you currently live?

____

☐8 I do not know/I am not sure.

☐9 I would rather not like to say.

What's your postal code?

☐1 ________________

☐8 I do not know/I am not sure.

☐9 I would rather not like to say.

What is your current marital status? *Select the box/category that best describes you.*

☐1 Married

☐2 Divorced

☐3 Widowed

☐4 Separated

☐5 Never married and not a member of an unmarried couple

☐6 Member of an unmarried couple

☐8 I do not know/I am not sure.

☐9 I would rather not like to say.

What is the highest grade or year of school you completed?

☐1 Never attended school or only attended kindergarten

☐2 Grades 1 through 8 (Elementary)

☐3 Grades 9 through 11 (Some high school)

☐4 Grade 12 or General Educational Development (GED) test (High school graduate)

☐5 College 1 year to 3 years (Some college or technical school)

☐6 College 4 years or more (College graduate)

☐8 I do not know/I am not sure.

☐9 I would rather not like to say.

What is your current employment status? *Select the category which best describes you.*

☐1 Employed for wages

☐2 Self-employed

☐3 Out of work for 1 year or more

☐4 Out of work for less than 1 year

☐5 Homemaker

☐6 Student

☐8 I do not know/I am not sure.

☐9 I would rather not like to say.

How do you manage on your available household income from all sources (if applicable, including social security, unemployment insurance and/or disability income)?

☐ 1 Easily

☐ 2 Not to bad

☐ 3 Difficult some of the time

☐ 4 Difficult all of the time

☐ 5 Impossible

☐ 8 I do not know/I am not sure.

☐ 9 I would rather not like to say.

How many people live in your household?

Number of adults (aged ≥ 18 years): ______________________________

Number of children (aged < 18 years): ______________________________

☐ 8 I do not know/I am not sure.

☐ 9 I would rather not like to say.

Would you say that in general your health is….

☐ 1 Excellent

☐ 2 Very good

☐ 3 Good

☐ 4 Fair

☐ 5 Poor

☐ 8 I do not know/I am not sure.

☐ 9 I would rather not like to say.

How many of your children are affected by a rare disease?

________ child/children

☐ 8 I do not know/I am not sure.

☐ 9 I would rather not like to say.

How many biological siblings does your affected child have?
________ siblings<br>☐8 I do not know/I am not sure.<br>☐9 I would rather not like to say.

Are you and the other parent of the affected child related? If yes, please indicate the degree.
☐1 Yes, degree:<br>☐2 No<br>☐8 I do not know/I am not sure.<br>☐9 I would rather not like to say.

Unmet needs and Experiences of parents of children with a rare disease[1]

[Parental needs for rare diseases][2]

Below are some needs that have been named by parents of a child with a rare disease. Please indicate your current support needs for each of the following questions. Please tick only one box per question. Tick the box that best describes you.

Part 1: Understanding the disease

How much support do you need with respect to the following?		Extremely Confident				I desperately need help
1.	Teaching my child about the disease	1	2	3	4	5
2.	Explaining my child´s disease to my parents or relatives	1	2	3	4	5

[1] Deutsche Übersetzung durch Prof. Beate Wild und Dr. Markus Haun, Klinik für Allgemeine Innere Medizin und Psychosomatik, Universität Heidelberg

[2] Pelentsov LJ, Fielder AL, Laws TA, Esterman AJ. Development of the parental needs scale for rare diseases: a tool for measuring the supportive care needs of parents caring for a child with a rare disease. *J Multidiscip Healthc*. 2016;9:425-433. Published 2016 Sep 9. doi:10.2147/JMDH.S113898.

Pelentsov LJ, Fielder AL, Laws TA, Esterman AJ. The supportive care needs of parents with a child with a rare disease: results of an online survey. *BMC Fam Pract*. 2016;17:88. Published 2016 Jul 21. doi:10.1186/s12875-016-0488-x.

3.	Responding when friends, neighbours, or others ask questions about my child	1	2	3	4	5
4.	Explaining my child´s disease to other children	1	2	3	4	5

Part 2: Working with health professionals

**How satisfied are you with the following:**		Extremely Satisfied				Extremely Dissatisfied
5.	Having a consistent team of health professionals taking overall responsibility for your child´s health	1	2	3	4	5
6.	The overall support that you get from health professionals for your child	1	2	3	4	5
7.	Feeling that you are part of a health care team looking after your child	1	2	3	4	5
8.	How much health professionals know about your child´s disease	1	2	3	4	5

Part 3: Financial Needs

**With respect to your child´s disease, how much financial assistance do you need with...:**		I can easily afford it				I cannot afford it
9.	Paying for medical care or therapy	1	2	3	4	5
10.	Paying for special equipment or clothing	1	2	3	4	5
11.	Paying for babysitting or respite care	1	2	3	4	5

Part 4: Informational, social, physical, spiritual, and psychological needs

**How much support do you need with respect to the following:**		Does not apply	Satisfied	Little need	Medium need	High need
12.	Information on the growth and development of my child	1	2	3	4	5
13.	Information on current services available for my child	1	2	3	4	5
14.	Information on future services available for my child	1	2	3	4	5
15.	Finding suitable childcare	1	2	3	4	5

16.	Reconciling work and family life	1	2	3	4	5
17.	Need to speak to other parents with similar experiences	1	2	3	4	5
18.	Relationship with my partner	1	2	3	4	5
19.	Relationship with my child´s siblings	1	2	3	4	5
20.	My insomnia	1	2	3	4	5
21.	My persistent tiredness	1	2	3	4	5
22.	My loss of appetite	1	2	3	4	5
23.	My feeling of physical exhaustion	1	2	3	4	5
24.	Finding meaning in the situation	1	2	3	4	5
26.	Speaking to health professionals	1	2	3	4	5
27.	Feeling useless, powerless, helpless	1	2	3	4	5

Do you have needs for any of the following services? *Please tick all boxes that apply to you.*

- ☐1 Marriage Counselling
- ☐2 Psychological Counselling
- ☐3 Financial Counselling
- ☐4 Support groups for parents
- ☐5 Genetic Counselling
- ☐6 Social Work
- ☐7 Others: ________________________________

Please tell us about any supportive care needs that you have which we have not covered in this questionnaire?
